# Impact of Mediterranean Diet on Lipid Composition in the Colaus-PsyColaus Study

**DOI:** 10.3390/nu15214659

**Published:** 2023-11-03

**Authors:** Mélisande Flatscher, Antoine Garnier, Pedro Marques-Vidal, Vanessa Kraege

**Affiliations:** 1 Faculty of Biology and Medicine, Lausanne University, 1011 Lausanne, Switzerland; 2 Medical Directorate, Lausanne University Hospital, 1011 Lausanne, Switzerland; antoine.garnier@chuv.ch (A.G.); vanessa.kraege@chuv.ch (V.K.); 3 Department of Medicine, Internal Medicine Division, Lausanne University Hospital, 1011 Lausanne, Switzerland; pedro-manuel.marques-vidal@chuv.ch; 4 Innovation and Clinical Research Directorate, Lausanne University Hospital, 1011 Lausanne, Switzerland

**Keywords:** mediterranean diet, lipids, dyslipidemia, nutrition, non communicable diseases, diet score

## Abstract

High adherence to the Mediterranean diet (MD) has been associated with lower incidence of cardiovascular disease, increased HDL-cholesterol levels, and decreased triglycerides (TG), and total and LDL cholesterol levels. We aimed to assess the association of MD adherence at baseline with the lipid profile both cross-sectionally and prospectively in a sample of apparently healthy community-dwelling subjects. We conducted three cross-sectional studies using data from follow-ups 1 (FU1, 2009–2012), 2 (FU2, 2014–2017), and 3 (FU3, 2018–2021) of CoLaus|PsyCoLaus, a population-based sample from Lausanne, Switzerland. Dietary intake was assessed with a food frequency questionnaire. Two MD scores (Trichopoulou and Vormund) were computed, ranging from 0 (low) to 9 (high). In total, LDL and HDL cholesterol and TG were assessed. Incident dyslipidemia was defined as hypolipidemic treatment at FU2 or FU3. Overall, 4249 participants from FU1 (53.7% women, 57.6 ± 10.5 years, Trichopoulou 4.0 ± 1.5, Vormund 4.7 ± 1.9) were included. Neither MD score correlated significantly with the lipid markers and similar results were obtained according to the hypolipidemic status. Among the 3092 untreated FU1 participants with FU2 and FU3 data, 349 (11.3%) developed dyslipidemia by FU2 or FU3. No difference in MD scores was found between participants who developed dyslipidemia and those who did not (4.1 ± 1.5 vs. 4.0 ± 1.5 and 4.8 ± 1.8 vs. 4.8 ± 1.9 for Trichopoulou and Vormund, respectively, *p* > 0.05). Finally, no associations were found between MD score and lipid changes at 5 or 10 years. Contrary to other studies, adherence to MD at baseline did not show any significant effects on lipid composition/incident dyslipidemia in Colaus|PsyCoLaus participants.

## 1. Introduction

Blood lipid composition has significant effects on cardiovascular health, more specifically on atherosclerotic cardiovascular disease (CVD) [1]. To improve cardiovascular health, low-density lipoprotein (LDL) cholesterol should be decreased below well-established goals, in combination with triglyceride (TG) reduction [1].

One way to improve lipid profile, and thus decrease incidence and mortality of CVD, is to improve life-style habits such as nutrition. The Mediterranean siet’s (MD) capacity to reduce CVD incidence has been documented by observational studies [2,3] and a large clinical trial [4]. For instance, Rosato et al.’s meta-analysis demonstrated an inverse relationship between a high adherence to a MD and the risk of coronary heart disease, acute myocardial infarction, ischemic stroke, or CVD mortality [5].

The MD is a traditional diet from areas of olive cultivation in the Mediterranean region, where it has been associated with better health status [6]. This food pattern is characterized by a high intake of plant foods such as fruits, vegetables, different forms of cereals, potatoes, beans, nuts, and seeds; a moderate to low intake of fish and dairy products with a moderate consumption of yogurt, cheese, poultry, and eggs; a low intake of red meat and sweets; and a low to moderate consumption of wine. The properties of this diet are based on the abundance of essential micronutrients, fibres, and antioxidants from plant foods, contrary to meat consumption. Olive oil is the primary source of fat and mainly provides monosaturated fat with low amounts of saturated fat. The benefits are derived from the synergy and addition of the different components of the MD, emphasizing the importance of assessing the whole diet rather than individual nutrients [3,5,7].

Most studies assessing the possible associations with adherence level to the MD reported a decrease in LDL, TG, and total cholesterol levels, and an increase in high-density lipoprotein (HDL) cholesterol [8,9,10,11,12,13]. Conversely, the results of randomized controlled trials on the effect of the MD on lipids were low to modest, or insignificant [14,15]. Thus, although most studies showed the same trend, the limitations are often numerous and proven beneficial effects of the MD are very small. In some cases, confounding factors limit the association between MD and lipid profile; healthy behaviours such as non-smoking, adequate physical activity or social interaction have a synergic effect with diet and the individual effect of the MD on lipid profile could not always be proven. [8,11,13]. In addition, the conclusions of some studies might not apply to the general population. For instance, some studies focused on people at high cardiovascular risk or with dyslipidaemia [12], while others mainly included healthy active men [11,13]. Other studies had small samples and thus reduced statistical power [9,11,12,14]. Finally, the cross-sectional design of some studies does not allow the drawing of temporal or causal associations, stressing the need for prospective studies with higher levels of proof [8,9,10,13].

We aimed to assess the effect of the MD on the lipid profile and its evolution both cross-sectionally and prospectively in a sample of apparently healthy community-dwelling subjects, independently of their lipid status.

## 2. Materials and Methods

### 2.1. Population and Study Design

The CoLaus|PsyCoLaus study is a population-based study investigating the epidemiology and genetic determinants of psychiatric and cardiovascular disease in Lausanne, Switzerland [16]. Briefly, a representative sample was collected through a simple, non-stratified random sampling of 19,830 individuals (35% of the source population) aged between 35 and 75 and of Caucasian origin. The initial recruitment and baseline study was conducted between June 2003 and May 2006. Three follow-ups resurveyed the whole cohort over a 15-year period, the first follow-up (FU1) was performed between April 2009 and September 2012 with a median follow-up time of 5.4 years (average 5.6, range 4.5–8.8); the second follow-up (FU2) was performed between May 2014 and April 2017 with a median follow-up time of 10.7 years (average 10.9, range 8.8–13.6); and the third follow-up (FU3) was performed between April 2018 and May 2021 with a median follow-up time of 14.5 years (average 14.6, range 13.2–17.3). Participants underwent similar interventions at baseline and at the three follow-ups, including an interview, clinical assessment, blood and urine sampling, and health and lifestyle questionnaires.

For this study, only data from the follow-up examinations were used because dietary assessment was not performed at baseline. Therefore, our detailed aims were to study the association between the MD score at FU1 and (1) lipid levels/hypolipidemic treatment at FU1; (2) incident dyslipidemia [defined as self-reported hypolipidemic treatment at FU2 or FU3] among untreated participants at FU1; and (3) changes in total LDL and HDL cholesterol between FU1 and FU2/FU3 among participants with/without statin treatment during the whole follow-up period.

### 2.2. Dietary Assessment

Dietary intake was assessed using a self-administered, semi-quantitative food frequency questionnaire (FFQ) which also included portion size [17]. This FFQ has been validated in the Geneva population [17,18]. Briefly, this FFQ assesses the dietary intake of the previous 4 weeks and consists of 97 different food items that account for more than 90% of the intake of calories, proteins, fat, carbohydrates, alcohol, cholesterol, vitamin D, and retinol, and 85% of fibre, carotene, and iron. To our knowledge, there is no FFQ (validated or not) assessing dietary intake for the whole year in Switzerland; the other available and validated FFQ assesses the dietary intake of the previous month [19]. Hence, this FFQ provides the best dietary assessment currently available. For each item, consumption frequencies ranging from “less than once during the last 4 weeks” to “2 or more times per day” were provided, and participants also indicated their average serving size (smaller, equal, or bigger) compared to a reference size. Each participant brought along their filled-in FFQ, which was checked for completion by trained interviewers on the day of the visit.

### 2.3. Quantification of the Mediterranean Diet Adherence Scores

Two dietary scores were computed based on the Mediterranean diet. The first Mediterranean dietary score (designated as ‘Mediterranean score 1′) was derived from Trichopoulou et al. [7] and uses consumption frequencies instead of amounts. Briefly, a value of 0 or 1 was assigned to seven different foods using their sex-specific medians as a cut-off. Participants whose consumption frequencies for “healthy” foods (vegetables, fruits, fish, and cereal) were above the median were given the value of 1, while for “unhealthy” foods (meat and dairy products), consumption frequencies below the median were given the value of 1. Two other items were considered, these were ratio of monounsaturated to saturated fats and moderate alcohol consumption (between 5 and 25 g/day for women and 10 and 50 g/day for men). The score ranged between 0 and 8.

The second Mediterranean dietary score (designated as ‘Mediterranean score 2′), adapted to the Swiss population, was computed according to Vormund et al. [20]. It used the same scoring system but considered nine types of “healthy” foods: fruits, vegetables, fish, cereal, salads, poultry, dairy products, and wine. The score ranged between 0 and 9. For both scores, higher values represented higher adherence to the MD and, hence, a healthier diet.

### 2.4. Lipids

Most biological assays were performed by the CHUV Clinical Laboratory on fresh blood samples within 2 h of blood collection. Lipid levels were determined by the following analytical procedures with maximum inter- and intra-batch coefficients of variability (CVs): total cholesterol by CHOD-PAP (cholesterol oxidase phenol 4-aminoantipyrine peroxidase) with 1.6–1.7% CVs; HDL-cholesterol by CHOD-PAP + PEG (polyethylene glycol) + cyclodextrin with 3.6–0.9% CVs; triglycerides by GPO-PAP (glycerol phosphate oxidase) with 2.9–1.5% CVs; and LDL particle size was assessed by polyacrylamide gel electrophoresis (Lipoprint LDL kit^®^, Quantimetrix Corporation, CA, USA) with 1.5–0.5% CVs.

Based on lipid concentration, the following definitions were used in this study: LDL-cholesterol was calculated with the Friedewald formula only if triglycerides <4.6 mmol/L; low HDL cholesterol level was defined as <1.0 mmol/L in men and <1.29 mmol/L in women; high HDL cholesterol as ≥1.6 mmol/L; high LDL cholesterol was defined as ≥4.1 mmol/L; and a high triglyceride level was defined as ≥2.2 mmol/L. Dyslipidaemia was defined as low HDL cholesterol and/or high triglyceride and/or LDL cholesterol ≥4.1 mmol/L or ≥2.6 mmol/L in the presence of a self-reported history of myocardial infarction, stroke, coronary artery disease, or diabetes.

Finally, participants were asked if they were known to have high cholesterol or were under hypolipidemic drug treatment.

### 2.5. Other Covariates

Data relative to demographic and socio-economic status, lifestyle factor, physical activity and food intake were collected through questionnaires. Marital status was categorized as living alone or as a couple; educational level was categorized as high (university), middle (high school), or low (mandatory or apprenticeship); smoking status was categorized as never, former, or current; physical activity was categorized as sedentary/active; sedentary status was defined as spending more than 90% of daily energy in activities below moderate- and high-intensity. Additional information on following other diets was collected, including weight loss, low fat, low sugar/diabetic, low salt, and other diets.

Body weight and height were measured with participants barefoot and in light indoor clothes. Body weight was measured in kilograms to the nearest 100 g using a Seca^®^ scale (Hamburg, Germany). Height was measured to the nearest 5 mm using a Seca^®^ (Hamburg, Germany) height gauge. Body mass index (BMI) was computed, obesity was defined as BMI ≥ 30 kg/m^2^ and overweight as BMI ≥ 25 and <30 kg/m^2^. Blood pressure (BP) was measured using an Omron^®^ HEM-907 automated oscillometric sphygmomanometer after at least a 10 min rest in a seated position, and the average of the last two measurements was used. Hypertension was defined by a systolic BP ≥ 140 mm Hg or a diastolic BP ≥ 90 mm Hg, or by the presence of antihypertensive drug treatment. Diabetes was defined as fasting plasma glucose ≥ 7.0 mmol/L and/or presence of oral hypoglycaemic or insulin treatment. Type 2 diabetes mellitus (T2DM) was defined in case of diabetes without self-reported Type 1 DM. Finally, baseline CVD was reported through interviews focused on personal and family history of CVD and cardiovascular risk factors.

### 2.6. Inclusion and Exclusion Criteria

We included participants who attended the first and at least one subsequent follow-up. We excluded those missing any variables required to define dietary assessment, lipid composition, or any covariables.

### 2.7. Statistical Analysis

The Shapiro–Wilk test was applied to test for normality. As the MD scores significantly deviated from a Gaussian distribution, the associations between MD scores and lipid levels at FU1 were assessed by Spearman’s nonparametric correlation. Adjustment for potential covariates was performed using linear regression and results were expressed as standardized beta coefficients. The association between MD scores and presence of hypolipidemic drug treatment upon FU1 was assessed by comparing the averages of MD scores between treated/untreated participants; bivariate and multivariable analyses were performed via analysis of variance (ANOVA).

To explore our second aim, to find the association between the MD scores and incident dyslipidemia, only participants devoid of hypolipidemic drug treatment at FU1 were included. We compared the MD scores at FU1 between participants who had started hypolipidemic drug treatment by FU2 or FU3 versus those who had not; bivariate and multivariable analyses were conducted using ANOVA. We also computed the incidence of hypolipidemic drug treatment according to MD levels at FU1. Analyses of the associations between the MD scores and incident hypolipidemic drug treatment as outcome were conducted using logistic regression.

Our third aim was to study the association between the MD scores and changes in total, LDL and HDL cholesterol between FU1 and FU2/FU3, among participants with/without statin treatment during the whole follow-up period. Participants who started hypolipidemic drug treatment after FU1 were thus excluded. We computed the difference in lipid levels between FU2 or FU3 and FU1 and associated this difference with the MD score at FU1. Analyses were conducted using Spearman’s correlation and multivariable linear regression, as was conducted for the first aim.

All multivariable analyses were adjusted for gender (man or woman), age (continuous), education (low, middle, or high), marital status (living alone or living in couple), smoking categories (never, former, or current), BMI categories (normal, overweight, or obese), hypertension (yes or no), and diabetes (yes or no).

Statistical analyses were conducted using Stata v.16.1 (Stata Corp, College Station, TX, USA) and statistical significance was considered for a two-sided test with *p* < 0.05.

## 3. Results

### 3.1. Selection and Characteristics of the Participants

**Figure 1:**
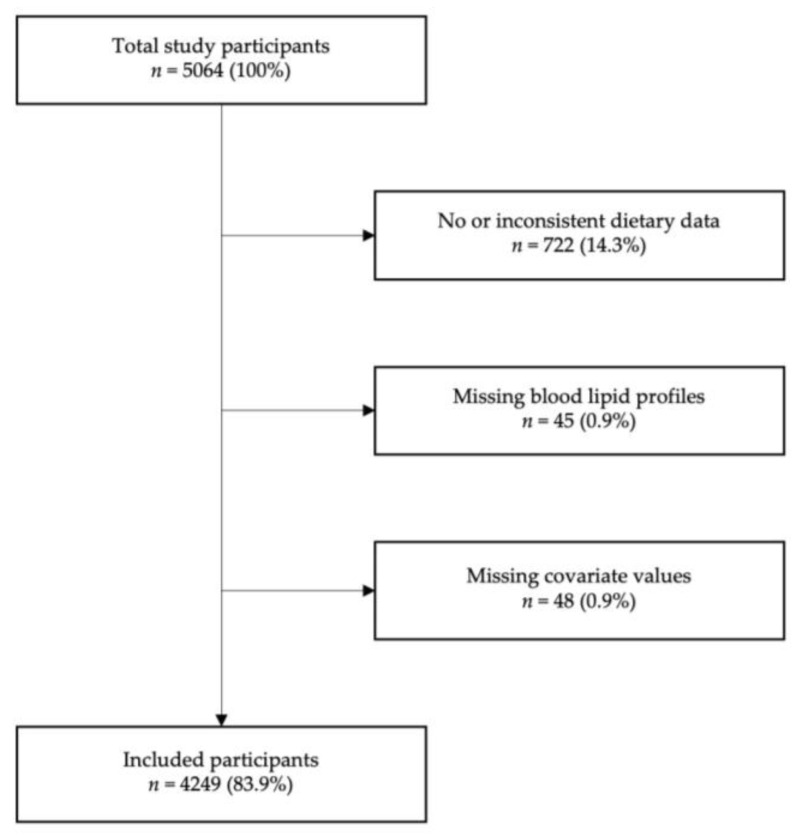
Exclusion criteria and number of included participants.

Of the 5064 participants who attended the first follow-up, 815 were excluded, leaving 4249 participants (83.9%) for analysis. Details of exclusions are indicated in Figure 1.

### 3.2. Mediterranean Diet Scores and Characteristics of the Participants

Characteristics of included and excluded participants from FU1 are presented in Table S1. Included participants were more often Swiss-born, non-smokers, living with someone, had higher educational status and MD scores, and were less likely to be obese, hypertensive, or diabetic.

### 3.3. Association between MD and Lipid Levels/Hypolipidemic Treatment at FU1

Table 1 shows the bivariate associations between both the MD scores and the lipid levels at the first follow-up according to presence or not of hypolipidemic treatment. Among participants untreated for dyslipidemia, MD scores were positively associated with HDL and negatively associated with triglyceride levels, while no association was found for total and LDL cholesterol. Among participants on hypolipidemic drugs, negative associations were found between the Vormund score and total and LDL cholesterol. Similar findings were obtained after multivariable analysis (Table S2).

### 3.4. Association between MD Scores and Incident Dyslipidemia

Among the 3485 participants with no hypolipidemic treatment at FU1, 3092 had follow-up data. Among these, 349 (11.3%) developed dyslipidemia upon FU2 or FU3. The characteristics of the participants according to absence or presence of incident dyslipidemia are detailed in Table 2. Participants newly treated for dyslipidemia were older, more often less educated, smokers, overweight, hypertensive, and diabetic. Conversely, there was no difference in either MD score between both groups. Similar negative findings were obtained after multivariable analysis taking into account age, gender, and other risk factors. The adjusted error for the Trichopoulou score 4.14 ± 0.08 vs. 4.00 ± 0.03 (*p* = 0.09) for participants with and without incident dyslipidemia, respectively, the corresponding values for the Vormund score being 4.94 ± 0.10 and 4.75 ± 0.04 (*p* = 0.07). When incident dyslipidemia was considered as the outcome, the multivariable-adjusted odds ratio and (95% confidence interval) for one unit increase in the Trichopoulou score was 1.06 (0.99–1.15), *p* = 0.10, and for one unit increase in the Vormund score 1.06 (0.99–0.12) *p* = 0.09.

### 3.5. Association between MD Scores and Changes in Lipid Levels between FU1 and FU2/FU3

The associations between the MD scores and lipid profile changes at 5-(FU2) to 10-year (FU3) follow-up stratified by statin treatment are indicated in Table 3. Among the participants untreated for dyslipidemia, changes in total cholesterol, LDL cholesterol, and triglycerides were positively associated with the Vormund scores. No significant consistent associations were found among participants treated for dyslipidemia. Furthermore, the correlation coefficients were low, indicating a weak association even in the case of significant lipid changes. Similar findings were obtained after multivariable linear regression adjusting for covariates (Table 4).

## 4. Discussion

### 4.1. Main Findings

Association of MD adherence with lipid markers was not significant in our cross-sectional study. The MD scores at baseline did not show any influence on dyslipidemia incidence upon follow-up, nor any prospective association with 5- to 10-year lipid changes.

### 4.2. Association of MD Score with Lipid Profile

The lack of association found in our study between MD adherence and lipid profile contrasts with previous cross-sectional studies (9–11.13). Higher MD scores have been associated with lower LDL cholesterol levels in 3775 Greek adults [10], with lower total and LDL cholesterol levels in 249 US firefighters [11] and with higher HDL cholesterol levels in 1290 Spanish adults [13]. Conversely, the effect of MD scores on triglyceride levels was inconsistent.

A first explanation might be the use of different scores among studies. Indeed, some studies included Mediterranean lifestyle aspects in addition to diet [11,13]. For instance, the MEDLIFE index includes dietary habits, physical activity, rest, and social interactions, in addition to the diet, as this would lead to a potentially greater synergistic effect than using the MD score alone [11].

A second hypothesis is the adaptation of MD scores to study populations, as stressed by Hoffman and Gerber [21]. Indeed, the Trichopoulou score was designed for a Greek population [7] and might not be adapted to other countries that differ in food availability and eating habits. In a study conducted in Belgium using the Trichopoulou score, only weak and inconsistent associations with the lipid profile were found [9]. To explore this second hypothesis in our study, we used a second Mediterranean diet score defined by Vormund and adapted to the Swiss population [20]. The main difference between this score and the one by Trichopoulou is the beneficial consumption of dairy products, which are highly consumed in Switzerland. Still, few significant associations were found, the Vormund score being associated with HDL cholesterol and TG in untreated participants, and with total and LDL cholesterol in treated participants.

### 4.3. Association of MD Score with Incident Dyslipidemia and Lipid Evolution

Our study did not confirm the negative association between MD adherence at baseline on the incidence of dyslipidemia or changes in lipid levels. According to a meta-analysis of randomized controlled trials [15], an MD intervention showed only moderate benefits on cardiovascular risk factors compared to no or minimal intervention. Among the studies included in that meta-analysis, nine studies on primary prevention showed low quality evidence for a small reduction in total cholesterol compared to baseline, and little or no effect on LDL, HDL cholesterol and triglycerides. Findings were similar for the two studies in this same meta-analysis assessing secondary prevention participants. Overall, our results suggest that adherence to a MD as defined by the Trichopoulou or Vormund score provides little protection against development of dyslipidaemia. Still, given the limitations of the MD scores, further studies should be conducted to confirm or inform our findings.

### 4.4. Strengths and Limitations

The main strength of our study is its prospective design to assess the evolution of lipid profile. The previous literature mainly assessed the association with MD through cross-sectional studies or less frequently through randomized controlled studies involving a shorter follow-up time. Moreover, we compared the associations between participants at both the primary and secondary prevention, which did not restrict the sample only to a healthy population.

Our study also includes several limitations. First, we only considered dietary information recorded at the first follow-up to measure adherence to the diet, hence the prospective part of our study only assessed the association of MD adherence at baseline with lipid evolution and incident dyslipidemia. Secondly, we used a Mediterranean diet score on a non-Mediterranean population. Despite the Vormund score putting this bias into perspective, neither score assesses quality of nutrients, behaviours or traditional Mediterranean lifestyle pattern. Thirdly, we could not adjust for other lifestyle factors such as physical activity levels, and, despite multivariable adjustment, we could not exclude the issue of residual confounding. Finally, participants were only composed of Caucasians living in an urban city. Indeed, a Swiss study showed significant differences in food consumption across the three main regions of Switzerland [22]. Similar limitations can be found when generalizing results to other countries due to the differences in nutrient intake, especially when considering a North–South gradient [23].

## 5. Conclusions

In contrast with the previous literature, adherence to a MD at baseline among the Colaus|PsyCoLaus participants was associated neither with lipid levels nor with incident dyslipidemia. Our results highlight the need for continued research on how to investigate long-term diet interventions across populations and question the one-diet-fits-all approach to meet a specific clinical outcome such as dyslipidemia.

## Figures and Tables

**Table 1:** Bivariate associations between Mediterranean diet scores and lipid levels in mmol/L, stratified by hypolipidemic treatment, first follow-up, 2009–2012, CoLaus|PsyCoLaus study, Lausanne, Switzerland.

	Trichopoulou	*p*-Value	Vormund	*p*-Value
Untreated (*n* = 3485)				
Total cholesterol	0.006	0.729	−0.015	0.387
LDL cholesterol	−0.006	0.705	−0.026	0.132
HDL cholesterol	0.044	0.009	0.071	<0.001
Triglycerides	−0.032	0.057	−0.076	<0.001
Treated (*n* = 764)				
Total cholesterol	−0.070	0.052	−0.073	0.043
LDL cholesterol	−0.065	0.071	−0.074	0.040
HDL cholesterol	0.041	0.253	−0.005	0.896
Triglycerides	−0.060	0.100	−0.036	0.324

Results are expressed as Spearman correlation coefficients.

**Table 2:** Characteristics of participants with or without incident hypolipidemic treatment at 5- or 10 years follow-up among untreated participants, CoLaus|PsyCoLaus study, Lausanne, Switzerland.

	No Incident Hypolipidemic Treatment	Incident Hypolipidemic Treatment	*p*-Value
Total	2743 (88.7)	349 (11.3)	
Woman (%)	1598 (58.3)	172 (49.3)	0.001
Age (years)	55.4 ± 10.0	59.4 ± 9.3	<0.001
Swiss-born (%)	1788 (65.2)	217 (62.2)	0.268
Educational level (%)			<0.001
High	684 (24.9)	64 (18.3)	
Middle	801 (29.2)	85 (24.4)	
Low	1258 (45.9)	200 (57.3)	
Living alone (%)	1149 (41.9)	150 (43.0)	0.697
Smoking status (%)			0.012
Never	1200 (43.8)	131 (37.5)	
Former	1021 (37.2)	130 (37.3)	
Current	522 (19.0)	88 (25.2)	
BMI (kg/m^2^)	25.3 ± 4.3	27.0 ± 4.5	<0.001
BMI categories (%)			<0.001
Normal	1441 (52.5)	119 (34.1)	
Overweight	970 (35.4)	159 (45.6)	
Obese	332 (12.1)	71 (20.3)	
Hypertension (%)	839 (30.6)	169 (48.4)	<0.001
Diabetes (%)	131 (4.8)	49 (14.0)	<0.001
Mediterranean diet score			
Trichopoulou	4.0 ± 1.5	4.1 ± 1.5	0.205
Vormund	4.8 ± 1.9	4.8 ± 1.8	0.402

BMI, Body mass index. Results are expressed as number of participants (column %) or as average ± standard deviation. Hypertension was defined as ≥140/90 mm Hg or presence of an antihypertensive drug treatment; diabetes was defined as fasting plasma glucose ≥ 7.0 mmol/L or presence of an antidiabetic drug treatment. Between-group comparisons performed using ANOVA.

**Table 3:** Bivariate associations between the Mediterranean diet scores at baseline and 5- to 10- year lipid changes, CoLaus|PsyCoLaus study, Lausanne, Switzerland; maximal difference taken into account.

	Trichopoulou	*p*-Value	Vormund	*p*-Value
Untreated (*n* = 2991)				
5- to 10-year changes				
Total cholesterol	0.001	0.941	0.052	0.005
LDL cholesterol	−0.000	0.995	0.043	0.020
HDL cholesterol	0.014	0.450	0.022	0.234
Triglycerides	0.021	0.245	0.038	0.036
Treated (*n =* 629)				
5- to 10-year changes				
Total cholesterol	0.020	0.625	0.030	0.453
LDL cholesterol	0.012	0.771	0.011	0.779
HDL cholesterol	−0.005	0.900	0.020	0.612
Triglycerides	0.040	0.319	0.071	0.076

Results are expressed as Spearman’s correlation coefficients.

**Table 4:** Multivariable analysis of the associations between the Mediterranean diet scores at baseline and 5- to 10- year lipid changes, CoLaus|PsyCoLaus study, Lausanne, Switzerland; maximal difference taken into account.

	Trichopoulou	*p*-Value	Vormund	*p*-Value
Untreated (*n* = 2991)				
5- to 10-year changes				
Total cholesterol	0.000	0.986	0.031	0.085
LDL cholesterol	−0.007	0.693	0.026	0.142
HDL cholesterol	0.022	0.238	0.026	0.167
Triglycerides	0.010	0.583	0.004	0.839
Treated (*n =* 629)				
5- to 10-year changes				
Total cholesterol	0.033	0.411	0.024	0.557
LDL cholesterol	0.040	0.328	0.130	0.750
HDL cholesterol	−0.017	0.678	0.000	0.987
Triglycerides	0.003	0.950	0.036	0.381

Results are expressed as multivariable-adjusted beta coefficients. Statistical analysis by linear regression adjusting for gender (man, woman), age (continuous), education (low, middle, high), marital status (living alone, living in couple), smoking categories (never, former, current), BMI categories (normal, overweight, obese), hypertension (yes, no), and diabetes (yes, no).

## Data Availability

The data of CoLaus|PsyCoLaus study used in this article cannot be fully shared as they contain potentially sensitive personal information on participants. According to the Ethics Committee for Research of the Canton of Vaud, sharing these data would be a violation of the Swiss legislation with respect to privacy protection. However, coded individual-level data that do not allow researchers to identify participants are available upon request to researchers who meet the criteria for data sharing of the CoLaus|PsyCoLaus Datacenter (CHUV, Lausanne, Switzerland). Any researcher affiliated to a public or private research institution who complies with the CoLaus|PsyCoLaus standards can submit a research application to research.colaus@chuv.ch or research.psycolaus@chuv.ch. Proposals requiring baseline data only, will be evaluated by the baseline (local) Scientific Committee (SC) of the CoLaus and PsyCoLaus studies. Proposals requiring follow-up data will be evaluated by the follow-up (multicentric) SC of the CoLaus|PsyCoLaus cohort study. Detailed instructions for gaining access to the CoLaus|PsyCoLaus data used in this study are available at www.colaus-psycolaus.ch/professionals/how-to-collaborate/ (accessed on 29 October 2023).

## Supplementary Materials

The following supporting information can be downloaded at: https://www.mdpi.com/article/10.3390/nu15214659/s1, Table S1: Characteristics of included and excluded participants at first follow-up, 2009–2012, CoLaus|PsyCoLaus study, Lausanne, Switzerland; Table S2: Multivariable analysis of the associations between the Mediterranean diet scores and lipid levels at baseline, stratified by hypolipidemic drug treatment, CoLaus|PsyCoLaus study, Lausanne, Switzerland.

## References

[1] Visseren F.L.J., Mach F., Smulders Y.M., Carballo D., Koskinas K.C., Bäck M., Benetos A., Biffi A., Boavida J.-M., Capodanno D. (2022). 2021 ESC Guidelines on cardiovascular disease prevention in clinical practice. Eur. J. Prev. Cardiol..

[2] Ahmad S., Moorthy M.V., Demler O.V., Hu F.B., Ridker P.M., Chasman D.I., Mora S. (2018). Assessment of Risk Factors and Biomarkers Associated with Risk of Cardiovascular Disease Among Women Consuming a Medi-terranean Diet. JAMA Netw. Open.

[3] Guasch-Ferré M., Willett W.C. (2021). The Mediterranean diet and health: A comprehensive overview. J. Intern. Med..

[4] Estruch R., Ros E., Salas-Salvadó J., Covas M.-I., Corella D., Arós F., Gómez-Gracia E., Ruiz-Gutiérrez V., Fiol M., Lapetra J. (2018). Primary Prevention of Cardiovascular Disease with a Mediterranean Diet Supple-mented with Extra-Virgin Olive Oil or Nuts. N. Engl. J. Med..

[5] Rosato V., Temple N.J., La Vecchia C., Castellan G., Tavani A., Guercio V. (2019). Mediterranean diet and cardio-vascular disease: A systematic review and meta-analysis of observational studies. Eur. J. Nutr..

[6] Willett W.C., Sacks F., Trichopoulou A., Drescher G., Ferro-Luzzi A., Helsing E., Trichopoulos D. (1995). Mediter-ranean diet pyramid: A cultural model for healthy eating. Am. J. Clin. Nutr..

[7] Trichopoulou A., Costacou T., Bamia C., Trichopoulos D. (2003). Adherence to a Mediterranean Diet and Survival in a Greek Population. N. Engl. J. Med..

[8] Platania A., Zappala G., Mirabella M.U., Gullo C., Mellini G., Beneventano G., Maugeri G., Marranzano M. (2018). Association between Mediterranean diet adherence and dyslipidaemia in a cohort of adults living in the Med-iterranean area. Int. J. Food Sci. Nutr..

[9] Mertens E., Mullie P., Deforche B., Lefevre J., Charlier R., Huybrechts I., Clarys P. (2014). Cross-sectional study on the relationship between the Mediterranean Diet Score and blood lipids. Nutr. J..

[10] Magriplis E., Panagiotakos D., Mitsopoulou A.-V., Karageorgou D., Bakogianni I., Dimakopoulos I., Micha R., Michas G., Chourdakis M., Chrousos G.P. (2019). Prevalence of hyperlipidaemia in adults and its relation to the Mediterranean diet: The Hellenic National Nutrition and Health Survey (HNNHS). Eur. J. Prev. Cardiol..

[11] Hershey M.S., Sotos-Prieto M., Ruiz-Canela M., Christophi C.A., Moffatt S., Martínez-González M.Á., Kales S.N. (2021). The Mediterranean lifestyle (MEDLIFE) index and metabolic syndrome in a non-Mediterranean working population. Clin. Nutr..

[12] Formisano E., Pasta A., Cremonini A.L., Di Lorenzo I., Sukkar S.G., Pisciotta L. (2021). Effects of a Mediterranean Diet, Dairy, and Meat Products on Different Phenotypes of Dyslipidemia: A Preliminary Retrospective Analysis. Nutrients.

[13] Peñalvo J.L., Oliva B., Sotos-Prieto M., Uzhova I., Moreno-Franco B., León-Latre M., Ordovás J.M. (2015). Greater Adherence to a Mediterranean Dietary Pattern Is Associated With Improved Plasma Lipid Profile: The Aragon Health Workers Study Cohort. Rev. Esp. Cardiol. Engl. Ed..

[14] Sotos-Prieto M., Ruiz-Canela M., Song Y., Christophi C., Mofatt S., Rodriguez-Artalejo F., Kales S.N. (2020). The Effects of a Mediterranean Diet Intervention on Targeted Plasma Metabolic Biomarkers among US Firefighters: A Pilot Cluster-Randomized Trial. Nutrients.

[15] Rees K., Takeda A., Martin N., Ellis L., Wijesekara D., Vepa A., Das A., Hartley L., Stranges S. (2019). Mediterra-nean-style diet for the primary and secondary prevention of cardiovascular disease. Cochrane Database Syst. Rev..

[16] Firmann M., Mayor V., Vidal P.M., Bochud M., Pécoud A., Hayoz D., Paccaud F., Preisig M., Song K.S., Yuan X. (2008). The CoLaus study: A population-based study to investigate the epidemiology and genetic de-terminants of cardiovascular risk factors and metabolic syndrome. BMC Cardiovasc. Disord..

[17] Bernstein M., Huot I., Morabia A. (1995). Amélioration des performances d’un questionnaire alimentaire se-mi-quantitatif comparé à un rappel des 24 heures. Santé Publique Vandoeuvre-Lès-Nancy.

[18] Beer-Borst S., Costanza M.C., Pechère-Bertschi A., Morabia A. (2009). Twelve-year trends and correlates of dietary salt intakes for the general adult population of Geneva, Switzerland. Eur. J. Clin. Nutr..

[19] Marques-Vidal P., Ross A., Wynn E., Rezzi S., Paccaud F., Decarli B. (2011). Reproducibility and relative validity of a food-frequency questionnaire for French-speaking Swiss adults. Food Nutr. Res..

[20] Vormund K., Braun J., Rohrmann S., Bopp M., Ballmer P., Faeh D. (2015). Mediterranean diet and mortality in Switzerland: An alpine paradox?. Eur. J. Nutr..

[21] Hoffman R., Gerber M. (2013). Evaluating and adapting the Mediterranean diet for non-Mediterranean populations: A critical appraisal. Nutr. Rev..

[22] Chatelan A., Beer-Borst S., Randriamiharisoa A., Pasquier J., Blanco J., Siegenthaler S., Paccaud F., Slimani N., Nicolas G., Camenzind-Frey E. (2017). Major Differences in Diet across Three Linguistic Regions of Swit-zerland: Results from the First National Nutrition Survey menuCH. Nutrients.

[23] Freisling H., Fahey M.T., Moskal A., Ocké M.C., Ferrari P., Jenab M., Norat T., Naska A., Welch A.A., Na-varro C. (2010). Region-Specific Nutrient Intake Patterns Exhibit a Geographical Gradient within and between European Countries. J. Nutr..

