# Supplementary material for: Impact of Mediterranean Diet on Lipid Composition in the Colaus-PsyColaus Study
Source: Nutrients. 2023 Nov 3;15(21):4659. doi: 10.3390/nu15214659 (PMC10650561; doi:10.3390/nu15214659)
Supplement: Supplementary file 1 [file nutrients-15-04659-s001.zip › nutrients-2675984-supplementary.pdf]

Supplementary information

Table S1. Characteristics of included and excluded participants at first follow-up, 2009-2012, CoLaus|PsyCoLaus study, Lausanne, Switzerland.

	Included	Excluded	P-value
Total	4,249 (83.9)	815 (16.1)	
Woman (%)	2,280 (53.7)	427 (52.4)	0.507
Age (years)	57.6 ± 10.5	58.5 ± 10.8	0.031
Swiss born (%)	2,770 (65.2)	414 (50.8)	<0.001
Educational level (%)			<0.001
High	941 (22.1)	138 (17.1)	
Middle	1,143 (26.9)	163 (20.1)	
Low	2,165 (51.0)	509 (62.8)	
Living alone (%)	1,763 (41.5)	439 (53.9)	<0.001
Smoking status (%)			<0.001
Never	1,745 (41.1)	290 (38.3)	
Former	1,634 (38.4)	249 (32.8)	
Current	870 (20.5)	219 (28.9)	
Body mass index (kg/m^2)	26.0 ± 4.5	27.3 ± 5.2	<0.001
BMI categories (%)			<0.001
Normal (< 25 kg/m^2)	1, 914 (45.0)	266 (35.5)	
Overweight (25.0-29.9 kg/m^2)	1, 657 (39.0)	303 (40.4)	
Obese (≥ 30.0 kg/m^2)	678 (16.0)	181 (24.1)	
Hypertension (%)	1,718 (40.4)	378 (47.0)	0.001
Diabetes (%)	411 (9.7)	128 (16.1)	<0.001
Mediterranean diet score			
Trichopoulou	4.0 ± 1.5	3.4 ± 1.3	<0.001
Vormund	4.7 ± 1.9	3.1 ± 2.0	<0.001

BMI, Body mass index. Results are expressed as number of participants (column %) or as average ± standard deviation. Hypertension was defined as ≥140/90 mm Hg or presence of an antihypertensive drug treatment; diabetes was defined as fasting plasma glucose ≥7.0 mmol/l or presence of an antidiabetic drug treatment. Between-group comparisons performed using ANOVA.

Table S2. Multivariable analysis of the associations between the Mediterranean diet scores and lipid levels at baseline, stratified by hypolipidemic drug treatment, CoLaus|PsyCoLaus study, Lausanne, Switzerland.

	Trichopoulou	P-value	Vormund	P-value
Untreated (*n* =3,485)				
Total cholesterol	0.000	0.986	-0.019	0.265
LDL cholesterol	-0.016	0.350	-0.029	0.088
HDL cholesterol	0.037	0.011	0.041	0.005
Triglycerides	-0.010	0.512	-0.041	0.011
Treated (*n* =764)				
Total cholesterol	-0.073	0.108	-0.057	0.109
LDL cholesterol	-0.079	0.027	-0.063	0.078
HDL cholesterol	0.030	0.361	-0.014	0.668
Triglycerides	-0.046	0.194	0.005	0.895

Results are expressed as multivariable-adjusted beta coefficients. Statistical analysis by linear regression adjusting for gender (man, woman), age (continuous), education (low, middle, high), marital status (living alone, living in couple), smoking categories (never, former, current), BMI categories (normal, overweight, obese), hypertension (yes, no), and diabetes (yes, no).
